# Evaluation of Pyrethroid Susceptibility in *Culex pipiens* of Northern Izmir Province, Turkey

**Published:** 2018-12-25

**Authors:** Onur Guntay, Mehmet Salih Yikilmaz, Huseyin Ozaydin, Savas Izzetoglu, Asli Suner

**Affiliations:** 1 Section of Molecular Biology, Department of Biology, Faculty of Science, Ege University, Izmir, Turkey; 2 Department of Biostatistics and Medical Informatics, Faculty of Medicine, Ege University, Izmir, Turkey

**Keywords:** Bioassay, Insecticide resistance, Pyrethroids, Piperonyl butoxide, *Culex pipiens*

## Abstract

**Background:**

Mosquitoes, being a nuisance species, are considered as one of the most important species in public health control programs due to their role as a vector in mosquito-borne diseases observed in humans and animals. We evaluated the susceptibility status of *Culex pipiens* collected from northern Izmir, Turkey in 2011–16.

**Methods:**

Mosquito larvae, collected from three different locations in northern İzmir, were reared in the laboratory. Adult susceptibility bioassays were performed using the WHO insecticide-impregnated papers including deltamethrin 0.05%, permethrin 0.75%, α-cypermethrin 0.05% and cyfluthrin 0.15%. In addition, adult bioassays were performed after the pre-exposure to piperonyl butoxide (PBO) to determine the contribution of P450 detoxification enzymes to the phenotypic resistance.

**Results:**

In all of the three populations, high levels of resistance were observed (mortalities<63%) to all of the four pyrethroids. Different pyrethroids but with the same mode of action can exhibit significantly different phenotypic resistance in a single population. PBO bioassays also showed that P450 detoxification enzymes can have diverse effects on different pyrethroids.

**Conclusion:**

Using just one chemical in a class of insecticide can be misleading for resistance studies.

## Introduction

More than 48 mosquito species have been identified in Turkey including *Culex pipiens*, is one of the major nuisances (1). *Culex* species are important vectors for lymphatic filariasis and many other viral diseases such as West Nile (2, 3). Since the 1950s, the control of pests has gained momentum with the inclusion of chemical compounds in the integrated pest management. Pyrethroids, which are in the recommended insecticides list of WHO for the adult mosquito control (4), are widely used in both indoor and outdoor in Turkey.

Long-term and frequent applications of the same insecticide may select resistant individuals and this selection pressure may eventually lead to resistant populations becoming established (4–6). Hence, monitoring of the resistance/susceptibility status of mosquito populations to insecticides is very important for the success of the vector-control programs.

The most common form of resistance to pyrethroids is called knock-down resistance (KDR), linked to a single nucleotide substitution in the *Vssc* gene encoding the voltage-sensitive sodium channel protein and involves reduced target-site sensitivity to pyrethroids (7, 8). The second common mechanism involved in pyrethroid resistance involves detoxification enzymes belonging to the three major enzyme families; namely, cytochrome P450 monooxygenases (CYPs), glutathione-S-transferases (GSTs) and carboxyl/cholinesterases (CCEs). These enzymes cause metabolic detoxification of pyrethroids before they reach their target site (9–11).

A few studies have reported the occurrence, distribution and some mechanisms involved in insecticide resistance among certain mosquito species throughout Turkey and in neighboring countries such as Greece and Iran (12–22). Yet, we have a very limited knowledge of the insecticide resistance status of mosquito populations in Turkey.

In this study, our aims were to determine resistance/susceptibility status of three natural populations of *Cx. pipiens* in northern Izmir, Turkey and to understand to role of P450 detoxification enzymes in different pyrethroids (deltamethrin, permethrin, α-cypermethrin and cyfluthrin).

## Materials and Methods

### Mosquito samples

Mosquito larvae were collected from three different field locations: Maltepe Village, Menemen (N38°37’06”, E26°53’25”) in 2011, Sasalı, Çiğli (N38°28’23”, E26°56’51”) and Ege University Botanical Garden, Bornova (N38°27’33”, E27°14’01”) in 2016. Sites were located in the same climatic area with a slight difference (the average winter and summer temperatures are 7.9 °C and 26.3 °C, respectively and the average annual rainfall is 687mm). Larvae were transported to the laboratory and reared under conditions at a temperature of 25 °C and 70% (±5%) RH with a 12:12h light: dark photoperiod.

### Preparation for morphological identification

Species identification based on morphology was performed by selecting 4th stage larvae from the three abovementioned populations. For the preparations, the method (23) has been used with slight modifications. *Culex pipiens* larvae are yellowish-brown in color and have long-medium thickness siphon (24). These characteristics were used for identification. Species determinations based on morphological keys (25–27) were completed using stereomicroscope with an installed Argenit camera (CAMERA M5 CMOS).

### Susceptibility bioassays

Adult bioassays were performed following WHO guidelines (WHO/VBC/81.80) using the WHO susceptibility test kits. All specimens used in the bioassay were at fixed ages (3–5d old) and non-blood fed female adults. Mosquitoes were exposed to filter papers impregnated separately with 0.05% deltamethrin (Batch No: DE 499, Expiry Date: Apr 2018), 0.75% permethrin (Batch No: PE 406, Expiry Date: Apr 2018), 0.05% α-cypermethrin (Batch No: AL 237, Expiry Date: Apr 2018), 0.15% cyfluthrin (Batch No: CY 123, Expiry Date: Apr 2018) and control (Batch No: PY 249, Expiry Date: Mar 2018) for 1h. In order to evaluate the P450 detoxification enzyme activity, 4% PBO-impregnated papers (Batch No: PB 019, Expiry Date: Mar 2018) were pre-exposed for 1h before deltamethrin and permethrin applications. This test was not carried out for α-Cypermethrin and Cyfluthrin applications. We have called this test as synergist bioassay test within this manuscript. Oil-impregnated papers were used for controls. All of the impregnated papers were obtained from WHO Vector Control Research Unit at Universiti Sains Malaysia. During the experiments, knock-down (KD) rates were recorded every 15min (28). After completing the exposure time, mosquitoes were gently transferred into plastic cups and provided with 10% sucrose solution for 24h. Experimental mortality was recorded after 24h recovery period.

For each test session, 4 batches of 20–25 mosquitoes and 2 batches of 20–25 mosquitoes were exposed to insecticide-impregnated and oil-impregnated papers, respectively (28). For each insecticide, three replicates were carried out and the results were pooled to obtain the mean value of each test. Temperature and humidity were maintained at 27 °C (±1), 80% (±10) RH throughout the bioassay and recovery period (28).

### Data analysis

Statistical analyzes of the bioassay results were performed using Kruskal-Wallis and Mann-Whitney U tests with the IBM SPSS ver. 21.0 (Chicago, IL, USA). The normal distribution of the data was examined using Shapiro-Wilk normality test. For all the hypotheses testing, an alpha of 0.05 was used as the cutoff for significance.

## Results

### Morphological identification

**Fig. 1.**
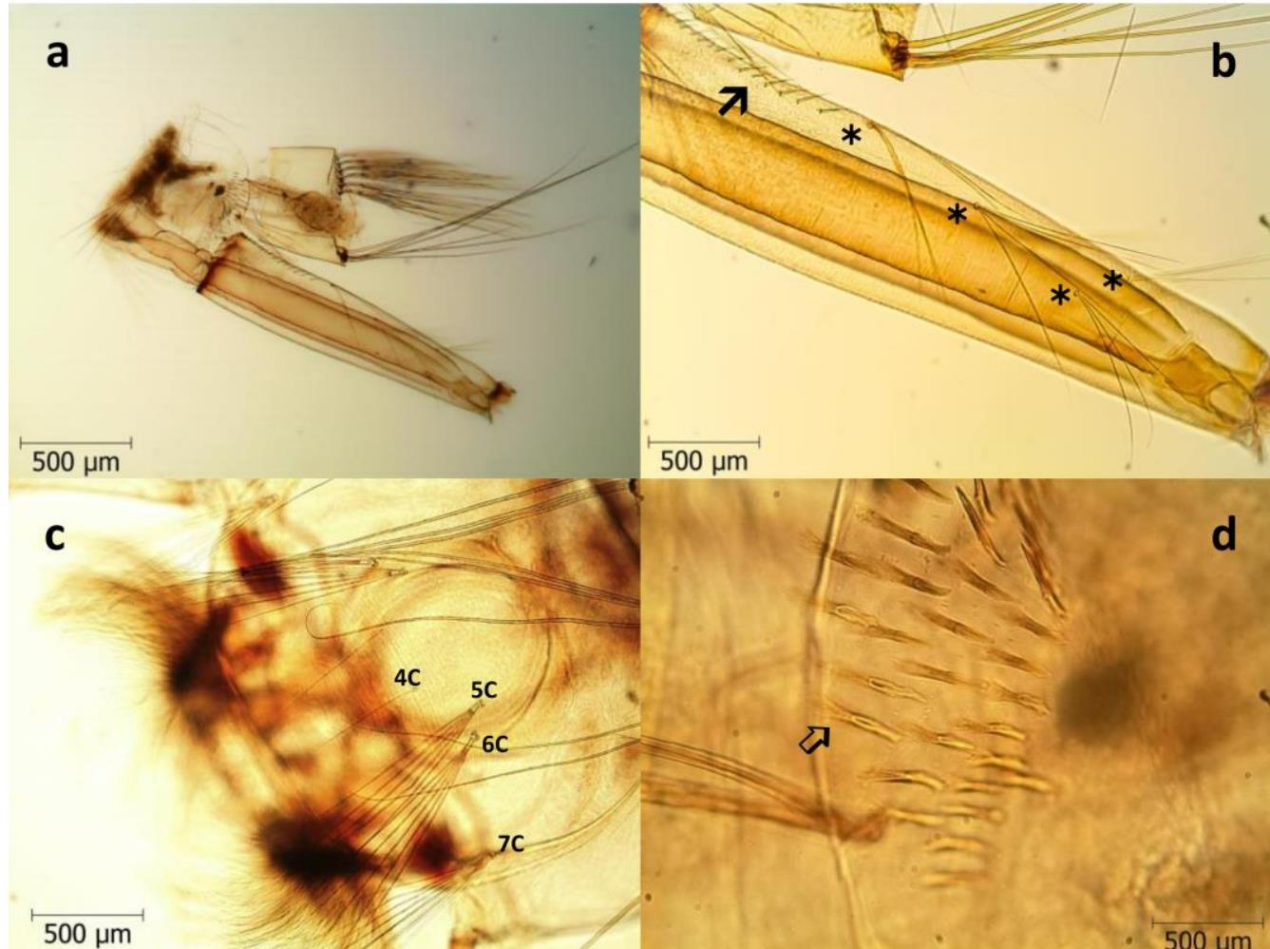
Some characteristics used in morphological identification. a) The last abdominal segment, anal segment and siphon of 4th stage larvae. b) Pecten teeth on siphon (shown by ⇗) and setae on siphon (all labeled with asterisk, *). c) Setae on the head; labeled as **4C–7C**. d) Combs on the last abdominal segment as shown by the arrow⇨

Some characteristics used for genus and species identification are shown in Fig. 1. Before applying bioassays, we identified that all populations collected were *Cx. pipiens*.

### Bioassays

**Fig. 2.**
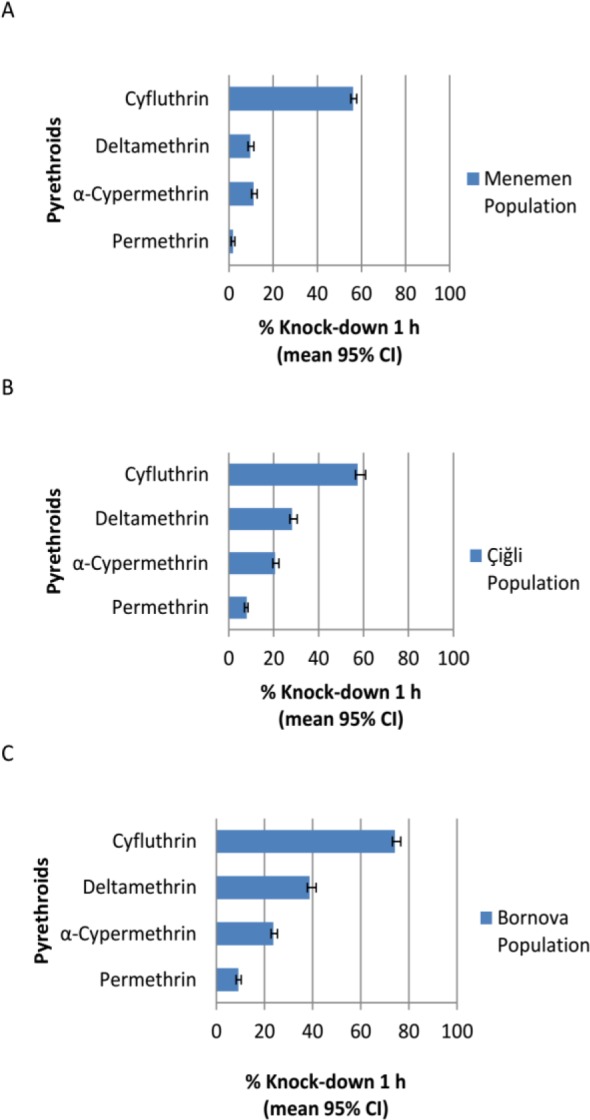
Knock-down rates of *Culex pipiens* at 1h of each tested population (A–C) in northern Izmir, Turkey, 2017

We have first analyzed the KD rates at the end of a 1h-application of each pyrethroid in each population. The pairwise comparisons of KD rates within the populations were tested for significance. Out of 18 pairwise comparisons (3 populations, 4 different pyrethroid applications, therefore 6 pairwise comparisons within each population), 7 were found to be significant (*P< 0.05). These significant results were obtained from the comparisons between the KD rates of Permethrin and Deltamethrin, Permethrin and Cyfluthrin, and α-Cypermethrin and Cyfluthrin applications in both Çiğli and Bornova populations. Moreover, one more significant result was also revealed from the pairwise comparison between KD rates of Deltamethrin and Cyfluthrin applications in Menemen population. The knock-down rates at 1h observed in all the populations of the study were summarized in Fig. 2.

All of the *Cx. pipiens* populations showed different levels of resistance to all tested insecticides with a mortality rate ranging between 0% and 63%. Overall, the lowest mortality rates were obtained with permethrin (ranged between 0% and 21%) and the highest mortality rates with cyfluthrin (ranged between 8% and 63%) in all populations. Mortality rates resulted from α-cypermethrin and deltamethrin applications ranged between 0% and 25% and between 0% and 38%, respectively. All the results obtained from susceptibility tests carried out in all of the three populations were summarized in Table 1.

**Table 1.** Mortality rate results (Izmir, Turkey, 2017) of permethrin (0.75%), α-cypermethrin (0.05%), deltamethrin (0.05%) and cyfluthrin (0.15%) tests obtained at 24h after the application in each of the tested *Culex pipiens* populations were presented below (95% CI)

**Populations**	**% Mortality (range)**			

	**Permethrin**	**α-Cypermethrin**	**Deltamethrin**	**Cyfluthrin**
**Menemen**	0.96 (0–4)	1.83 (0–9)	1.24 (0–7)	17.97 (8–29)
**Çiğli**	2.52 (0–7)	2.59 (0–8)	13.12 (3–22)	27.42 (14–46)
**Bornova**	8.15 (0–21)	11.39 (0–25)	16.98 (0–38)	46.27 (36–63)

Mortalities observed in cyfluthrin applications were significantly different than the mortality rates obtained from the other three pyrethroids used in the study in both Menemen and Bornova populations (*P< 0.05). For Çiğli population, we have found statistically significant difference (*P< 0.05) between mortality rates of cyfluthrin and permethrin applications as well as that of between cyfluthrin and α-Cypermethrin applications. However, there was no significant difference (P> 0.05) between mortality rates of cyfluthrin and deltamethrin applications. In addition, there was no significant difference between mortality rates of permethrin and α-Cypermethrin applications in Çiğli population. Furthermore, the pairwise comparison of mortality rates of Permethrin, α-Cypermethrin and deltamethrin applications in Bornova and Menemen populations, separately, did not reveal a significant difference.

The synergist bioassay test carried out in Menemen and Bornova populations in order for the assessment of the contribution of P450 detoxification enzymes did not restore susceptibility to permethrin and deltamethrin. However, in both populations, pre-exposure to PBO significantly increased the effect of the pyretroids applied. In Menemen population, the mortality rates significantly (*P< 0.05) increased from 1.24% (deltamethrin) to 23.96% (PBO+ deltamethrin) (estimated P= 0.000) and from 0.96% (permethrin) to 4.26% (PBO+ permethrin) (estimated P= 0.014). Similarly, in Bornova population, mortality rates significantly (*P< 0.05) increased from 16.98% (deltamethrin) to 55.96% (PBO+deltamethrin) (estimated P= 0.000) and from 8.15% (permethrin) to 27.95% (PBO+permethrin) (estimated P= 0.000). This bioassay was not carried out in Çiğli population. The comparative results of the synergist bioassays were summarized in Table 2.

**Table 2.** Susceptibility test results of *Culex pipiens* with and without pre-exposure to PBO in northern Izmir, Turkey, 2017 (95% CI)

**Populations**	**Insecticide tested**	**% Mortality (range)**
**Menemen**	Deltamethrin	1.24 (0–7)
	PBO+Deltamethrin	23.96 (8–35)
	Permethrin	0.96 (0–4)
	PBO+Permethrin	4.26 (0–9)
**Bornova**	Deltamethrin	16.98 (0–38)
	PBO+Deltamethrin	55.96 (37–68)
	Permethrin	8.15 (0–21)
	PBO+Permethrin	27.95 (16–50)

## Discussion

*Culex pipiens* is the most common mosquito in Turkey and mosquito control is mainly directed against larval stages besides, many pyrethroids are currently registered and used for adult control (15). Many pesticides commonly used in agricultural areas and public health cause mosquito populations to become resistant. The insecticides with same mode of action used in these different areas cause cross-resistance (29, 30).

Resistance was expected in these populations due to frequent use of agricultural pesticides in the vicinity of the settlement areas where populations were collected. Nevertheless, some mortality rates for permethrin, deltamethrin and α-cypermethrin were 0%, and therefore the detection of such a high resistance was surprising. Resistance to permethrin and deltamethrin was also detected in *Cx. pipiens* populations collected from similar regions in 2012 and 2013, but such low mortality rates have not been determined (18). In our study, the overall mortality rate for deltamethrin was 17%. In contrast, value of this species for deltamethrin in northwestern and southeastern Iran reported as 91% and 93% respectively (21, 22). Nevertheless, in Tehran, mortality rate for deltamethrin in same species was reported as 18% (20). In Greece, the lowest mortality rate was reported 64% in 13 different populations of the same species (19).

Different mortality rates can occur within different pyrethroids with the same mode of action (31). However, in the same population, the difference between cyfluthrin and other pyrethroid mortality means was not expected to be as high as 38% (Table 1). This may be related to the chemical structure of the pyrethroid used. The presence of the α-cyano group in the structure of the pyrethroid has an effect on the resistance mechanism(s) (32). This difference can also be explained by the reflection of different kdr mutations in phenotypic resistance in different ways (33). A possible third explanation is the different detoxification enzymes involved in metabolic resistance mechanisms. CYPs are major mechanism of insecticide metabolic resistance (34). Previous years, different results have been obtained about the role of GSTs and CCEs in pyrethroid resistance (10, 35, 36). Under different pyrethroid pressures, metabolic resistance mechanisms can change and phenotypic resistance can reflect differently. Under low pyrethroid selection pressure, metabolic resistance is mainly mediated by CYPs, but under high pyrethroid selection pressure, high level of metabolic resistance is related to CYPs and CCEs (11).

In this study, we used PBO, the CYPs inhibitor (37, 38) to demonstrate the effects of CYPs in metabolic resistance of deltamethrin and permethrin. Our results showed that CYPs indeed play a role in the metabolic resistance of both deltamethrin and permethrin (Table 2). In Menemen and Bornova populations, synergistic value between permethrin and PBO+ permethrin was 3.3% and 19.8% respectively. Synergistic results between same pyrethroid and PBO reported in two different population of same species in Marin County, California as 55.5% and 12.8% respectively (39).

Interestingly, in both populations with pre-exposure PBO, the effect of CYPs was higher in deltamethrin than in permethrin. Besides the detoxification enzymes, the physical properties and chemical structures of the insecticide used may also play a role together in metabolic resistance mechanisms.

## Conclusion

*Culex pipiens* mosquitoes collected from the three different localities in northern Izmir have high levels of resistance to permethrin, deltamethrin, α-cypermethrin and cyfluthrin. In additions, P450 detoxification enzymes have an effect on phenotypic resistance. However, the major mechanism is due to the kdr resistance. Further studies are needed to explain these mechanisms responsible for pyrethroid resistance in *Cx. pipiens*. The resistance found in this study is highly likely to be caused by pyrethroid spraying in agricultural areas. If some measures are not taken, these resistant populations will increase and lead to serious problems in public health in Izmir.
